# The Influence of Synthesis Parameters on the Properties of Dextran-Based Hydrogels for Colon-Targeted Antitumor Drug Delivery Part I: Room Temperature Synthesis of Dextran/Inulin Hydrogels for Colon-Targeted Antitumor Drug Delivery

**DOI:** 10.3390/gels11121011

**Published:** 2025-12-16

**Authors:** Tamara Erceg, Miloš Radosavljević, Milorad Miljić, Aleksandra Cvetanović Kljakić, Sebastian Baloš, Katarina Mišković Špoljarić, Ivan Ćorić, Ljubica Glavaš-Obrovac, Aleksandra Torbica

**Affiliations:** 1 Faculty of Technology Novi Sad, University of Novi Sad, Bulevar Cara Lazara 1, 21000 Novi Sad, Serbia; milos1506@gmail.com (M.R.); a.c.istrazivac@gmail.com (A.C.K.); 2 Institute of Food Technology in Novi Sad, University of Novi Sad, Bulevar Cara Lazara 1, 21000 Novi Sad, Serbia; milorad.miljic@fins.uns.ac.rs (M.M.); aleksandra.torbica@fins.uns.ac.rs (A.T.); 3 Faculty of Technical Sciences, University of Novi Sad, Trg Dositeja Obradovića 6, 21000 Novi Sad, Serbia; sebab@uns.ac.rs; 4 Faculty of Medicine, Josip Juraj Strossmayer University of Osijek, Josipa Huttlera 4, 31000 Osijek, Croatia; kmiskovic@mefos.hr (K.M.Š.); icoric@mefos.hr (I.Ć.); lgobrovac@mefos.hr (L.G.-O.)

**Keywords:** dextran, inulin, hydrogel, colon-specific drug delivery

## Abstract

This research successfully developed novel hydrogels composed of methacrylated dextran and inulin for targeted drug delivery in colorectal cancer therapy. The formulation exploits the natural degradation of both biopolymers by the large intestine’s microflora. A key achievement was the development of a room-temperature free radical polymerization synthesis method. The study thoroughly investigated how varying inulin content (10 and 20 wt%) influenced the hydrogels’ properties. The formulation with 20 wt% inulin exhibited the highest swelling ability at both pH 3 and pH 6, and consequently the lowest elastic modulus, measured by a newly established technique for granulated hydrogels. Using uracil as a model drug, in situ incorporated, confirmed that the greatest drug release occurs in the colorectal region for the neat dextran-based hydrogel, triggered by specific microbial enzymes. Notably, the addition of inulin did not enhance biodegradation-driven drug release in combination with dextran; instead, inulin primarily acted as a protective component against premature hydrolysis in the gastric medium. These findings strongly confirm that the targeted action is predominantly governed by the dextran component. The synthesized hydrogels, particularly the dextran-only formulation, therefore show strong potential as effective carriers for colon-targeted drug delivery. The primary objective of this study was to evaluate the feasibility of modified and unmodified dextran and inulin as biodegradable carriers for enzyme-triggered, colon-targeted drug delivery.

## 1. Introduction

The therapeutic effect of a drug, especially in the challenging context of tumor treatment, is strongly influenced by the drug carrier. The carrier role is crucial in antitumor treatment due to its ability to considerably improve therapeutic efficacy while minimizing side effects associated with conventional treatments. The primary advantage of drug carriers in cancer therapy lies in their ability to precisely target tumors, using passive mechanisms such as the enhanced permeability and retention (EPR) effect or active targeting via ligands that bind to specific cancer cell receptors [1]. Carriers protect drugs from premature action and degradation by encapsulating them, thereby extending their half-life in the bloodstream and improving their ability to reach the target site. They also enable the controlled release of therapeutics, either continuously or in response to specific stimuli from the tumor microenvironment, the presence of specific enzymes, or external stimuli such as light or a magnetic field. Additionally, carriers help overcome drug resistance that cancer cells can develop to chemotherapy through the mechanism of drug efflux, which involves pumping the drugs out of the cells. This is achieved by optimizing their size to the nanoscale level, thereby facilitating drug penetration into the cell and resulting in higher intracellular concentrations [2,3]. Finally, the versatility of the carrier supports combination therapy, allowing for synergistic effects with potentially lower toxicity of individual drugs [4]. Hydrogels are promising materials as carriers, representing a three-dimensional hydrophilic polymer network that can successfully trap drug molecules under mild conditions, enabling their controlled delivery to a specific location in the body [5]. Their design allows them to utilize both passive and active targeting mechanisms to improve drug delivery. Hydrogel carriers offer an advanced approach to controlled drug delivery by utilizing stimuli-responsiveness, a mechanism where drug release is triggered by specific environmental cues. This design allows the hydrogels to remain intact until they encounter environmental triggers such as a certain pH value or the presence of specific enzymes capable of degrading their polymer structure. Biopolymer-based hydrogels possess additional desirable properties, such as biocompatibility, biodegradability, low toxicity, as well as the ability to be shaped to simulate the extracellular matrix [6]. As natural biopolymers, polysaccharides can meet these requirements. Due to the numerous hydroxyl groups in their structure, carboxylic groups, and amino groups, these molecules make a strong platform for the preparation of hydrogels via chemical and physical crosslinking. Chemical crosslinking is more desirable, providing permanent hydrogels with the reversible ability to uptake and deliver environmental media. For the entrapping of active compounds, the in situ method is the most suitable, providing high encapsulation efficiency. Different antitumor drug-loaded polysaccharide-based hydrogels, mostly obtained in mild conditions using physical methods of crosslinking (ionic crosslinking), are investigated in the treatment of cancer of different organs. Hyaluronic acid-doxorubin conjugate has been investigated in the treatment of breast cancer, categorized as a leading cancer type [7]. The second most deadly type of cancer is colorectal cancer. Alginate/carboxymethyl cellulose-based hydrogels obtained by ionic crosslinking have been investigated in the treatment of this cancer type [8]. Sodium alginate-based hydrogels crosslinked with divalent and trivalent cations, such as calcium and iron, have been investigated as carriers for anticancer drug delivery by different research groups [9,10]. These systems function as long-term drug “depots,” enabling sustained and controlled release due to their bulk, microsized, physically crosslinked hydrogel structure, which represents one of the most widely applied depot-type delivery platforms reported in the literature. This type of hydrogel, due to the ionic groups, represents pH-responsive drug delivery. However, this drug release mechanism is not particularly reliable due to the large variation in the pH value of different parts of the digestive tract and the influence of various factors on this value, which results in the pH-sensitive carrier not directing the drug to the target site [11]. Considering the specific microenvironment of the colorectal region, influenced by the presence of the colon’s microflora, it is very useful for targeted delivery to the colon to obtain carriers that release entrapped drugs by biodegradation under specific enzymes. These biopolymers are selectively degraded by specific colonic bacterial enzymes—dextranase, amylase, and inulinase, respectively—ensuring targeted release. This dual-enzyme targeting creates a synergistic release mechanism. This possibility is given by the preparation of hydrogels based on dextran and inulin. Oxidized dextran-based stimuli-sensitive nanogel with covalently conjugated doxorubicin developed via Schiff base formation using the inverse microemulsion technique has also been investigated as a potential drug delivery system [12]. Hovgaard and Brøndsted have synthesized dextran-based carriers cross-linked with aliphatic diisocyanate, which are biodegradable in a human colonic fermentation model [13,14]. Numerous studies have confirmed the ability of inulin hydrogels to serve as a targeted delivery system for antitumor drugs to the colon. To obtain an inulin-based hydrogel with low swelling ability and high resistance in acidic media, Tripodo et al. modified inulin with methacrylic anhydride, derivatized it with succinic acid, and crosslinked it using UV irradiation. The model drug is incorporated by soaking [15]. In another study, hydrogels were synthesized through the esterification of inulin with pyromellitic dianhydride at room temperature in dimethylformamide [16]. Vervoort, with coauthors, also prepared inulin derivatized with glycidyl methacrylate in dimethyl formamide (DMF) at room temperature. Hydrogels based on this methacrylated inulin were subsequently prepared at room temperature in an aqueous medium. Their in vitro investigation focused on the targeted release of proteins to the colon, demonstrating the influence of both the degree of substitution and loading conditions on the release kinetics [17,18]. In a study conducted by Maris et al., hydrogels were created from methacrylated inulin (IN-MA), which was then copolymerized with bis(methacryloylamino)azobenzene (BMAAB), an aromatic azo agent, and either 2-hydroxyethyl methacrylate (HEMA) or methacrylic acid (MA) [19].

Despite the growing interest in polysaccharide-based hydrogels for biomedical applications, a significant research gap exists regarding hydrogels synthesized from a combination of dextran and inulin. The development of a new formulation of dextran and inulin is based on their complementary physicochemical and enzymatic properties, to overcome degradation in the acidic environment of the stomach and achieve targeted release in the colon. Dextran is an excellent material for drug delivery to the colon due to its ability to carry various substances, biocompatibility, and, most importantly, its degradation by intestinal microflora enzymes (dextranase). This enables the targeted release of the drug exactly where it is needed. However, the main challenge lies in its sensitivity to gastric acidity, where low pH values (around 1.5–3.5) can break its glycosidic bonds (α-(1→6), α-(1→3), α-(1→4), or α-(1→2)) [20,21]. This potentially can lead to the premature release of the drug in the stomach, which causes it to be lost and the entire system to lose its functionality. To mitigate this, inulin was incorporated as a critical functional excipient for three reasons: first, its β-(2→1) fructan bonds are resistant to acid hydrolysis, so in the stomach the inulin matrix remains stable and protects dextran and the encapsulated drug from premature degradation; secondly, both polymers are targeted for degradation by colonic enzymes—dextranase and inulinase—which provides a synergistic drug release mechanism that remains inert in the upper gastrointestinal tract and is efficiently activated in the colon; third, as a well-known prebiotic, inulin promotes the growth of beneficial bacteria (*Bifidobacteria* and *Lactobacilli*), potentially increasing the populations of microbes that produce the relevant enzymes and thus further enhancing the effectiveness and specificity of the release trigger [22,23].

Our comprehensive investigation addresses this void by systematically exploring the influence of hydrogel composition on their swelling, morphological, structural, and mechanical properties, giving insight into the behavior of hydrogels under the influence of different enzymes, present in the digestive tract, using uracil as a safe active compound due to its structural similarity to the commercial antitumor drug 5-fluorouracil. The objective of this work is to optimize an energy-efficient, room-temperature synthesis method for dextran/inulin hydrogel preparation, which facilitates the efficient encapsulation of thermolabile antitumor molecules, avoiding reported complex procedures for carrier synthesis. This development is further complemented by the introduction of a novel methodology for characterizing the mechanical properties of granulated hydrogels, providing a robust tool for future material design, as well as by the establishment of a scalable procedure for the preparation of hydrogels in granulated form for colorectal cancer treatment. This dosage form is intended for administration in capsules, thereby preventing premature dextran hydrolysis and undesired drug release under the acidic conditions of the gastric environment. To the best of our knowledge, no closely related studies combining enzyme-triggered colon targeting, granulated hydrogel mechanics, and scalable room-temperature synthesis of dextran/inulin systems have been reported to date.

## 2. Results and Discussion

### 2.1. Results of FTIR Analysis

**Figure 1:**
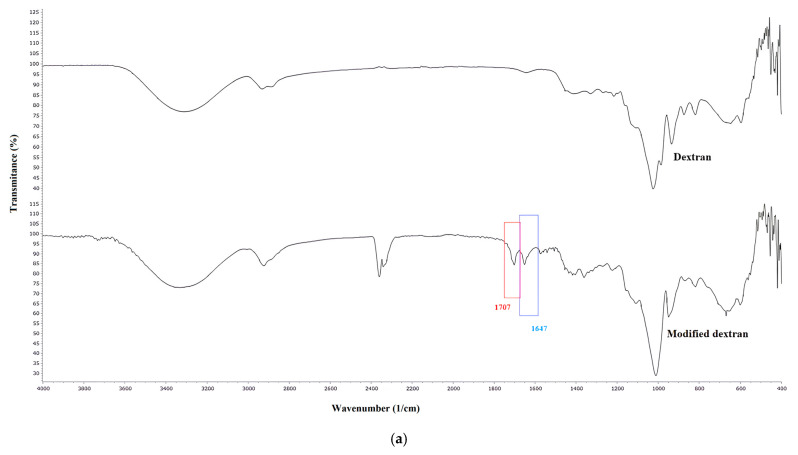

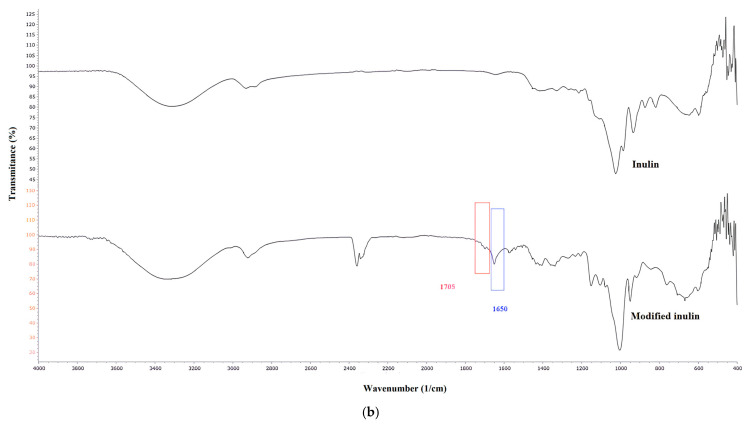
FTIR spectra of: (**a**) dextran and modified dextran (dextran methacrylate), (**b**) inulin and modified inulin (inulin methacrylate).

Figure 1 compares the FTIR spectra of dextran and modified dextran (Dextran-MA) (Figure 1a), as well as of inulin and modified inulin (Inulin-MA) (Figure 1b). A broad band with the peak at 3300 cm^−1^ present in all spectra is attributed to the stretching vibration of the OH group. Two peaks between 2930 and 2800 cm^−1^ are assigned to the -CH stretching vibrations. Bands at 1707 and 1647 cm^−1^ in FTIR spectra of dextran and modified dextran (Figure 1a) are assigned to the carbonyl group from the ester functionality and the carbon–carbon double bonds of the conjugated system, respectively. Modification of inulin has been confirmed by the presence of the same bands at 1705 and 1650 cm^−1^ (Figure 1b). This confirms successful modification of dextran and inulin by glycidyl methacrylate. Two peaks between 1420 and 1335 cm^−1^ in all spectra correspond to CH_2_ and CH_3_ bending. A peak at 1154–1157 cm^−1^ is attributed to the C-O-C stretching vibration. The absorption bands in the range 1080–1015 cm^−1^ are attributed to C-C-O stretching and C-O-H bending. Peaks at 915–800 cm^−1^ in FTIR spectra of all samples are assigned to the glycosidic bond.

**Figure 2:**
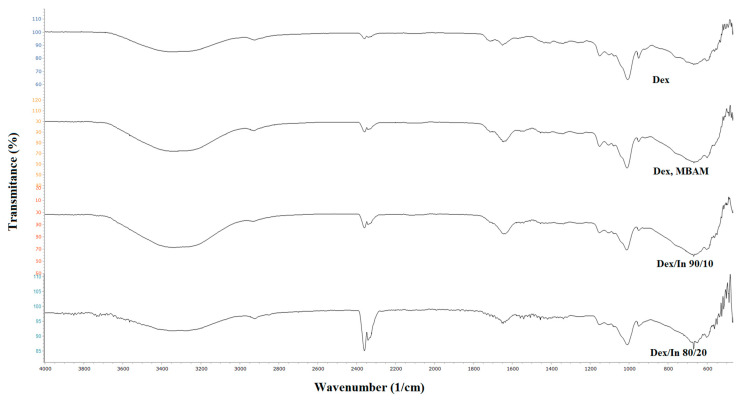
FTIR spectra of hydrogels.

The obtained hydrogels all have very similar FTIR spectra (Figure 2). Analysis of these spectra revealed a shift in the peak, corresponding to the carbonyl group, to higher wavenumbers, specifically within the range of 1715–1720 cm^−1^. Additionally, peaks attributed to the glycosidic bond were observed at 951 cm^−1^ and 919 cm^−1^.

**Figure 3:**
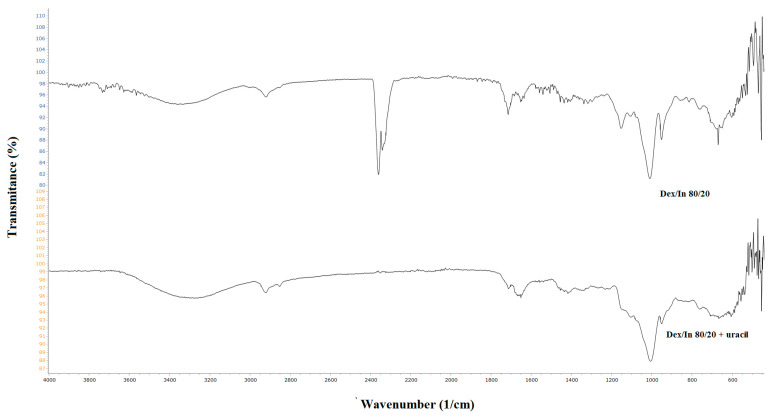
FTIR spectra of Dex/In hydrogel and uracil-loaded Dex/In 80/20 hydrogel.

After the incorporation of uracil into the structure of Dex/In 80/20 hydrogel, FTIR spectra were recorded (Figure 3). Overlapping peaks from both the hydrogel and the drug are observed, such as the N-H group from uracil and the O-H group from the dextran-based hydrogel. An absorption band corresponding to the C-O-H bending vibration is shifted to a lower wavenumber of 1006 cm^−1^, indicating a change in the molecular environment as a result of the drug’s presence within the polymer matrix and the presence of hydrogen bonds between amide groups of the crosslinking agent (MBAM), and hydroxyl and ester groups of modified dextran and inulin.

### 2.2. Results of DSC Analysis

**Figure 4:**
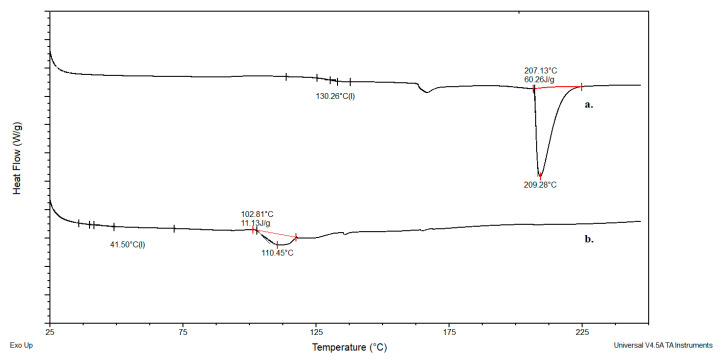
DSC curves for: a. dextran, b. modified dextran.

**Figure 5:**
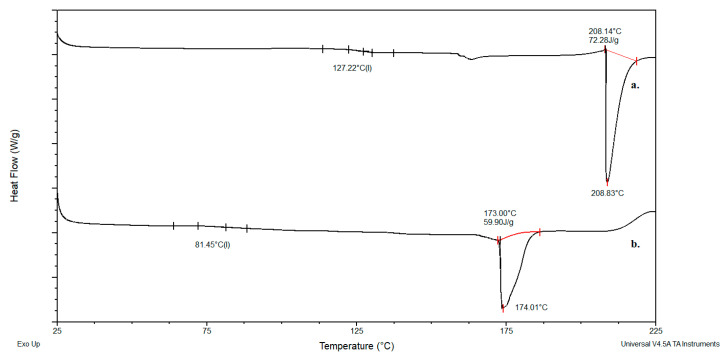
DSC curves for: a. inulin, b. modified inulin.

Figure 4 presents the DSC thermograms for neat dextran and dextran modified with glycidyl methacrylate. The methacrylated dextran exhibits a glass transition temperature of 41 °C and a melting point of 110 °C, while the neat (unmodified) dextran shows a glass transition temperature of 130 °C and a melting point of 209 °C. Similarly, Figure 5 displays the DSC thermograms for neat inulin and methacrylated inulin. Neat inulin demonstrates a glass transition temperature of 127 °C, and a melting point of 208 °C, whereas the modified inulin exhibits a glass transition temperature of 81 °C, and melts at 174 °C. The observed reduction in glass transition temperature is a consequence of improved segmental mobility resulting from the voids between chains introduced by the incorporation of methacrylate groups. A reduction in melting enthalpy (from 60.2 to 11.13 J/g for the dextran and 72.28 to 59.90 J/g for inulin) and temperature is a consequence of disrupting the original structure of the biopolymer, introducing imperfections, and thereby reducing the total energy required to transition the material from a solid to a liquid state.

### 2.3. Results of SEM Analysis

**Figure 6:**
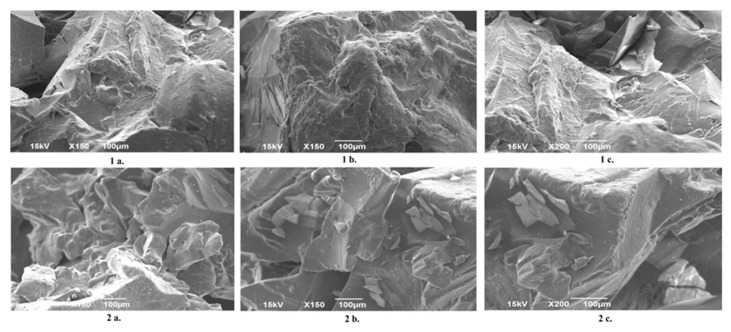
SEM images of neat Dex/In 80/20 (**1a**–**c**) and drug-loaded Dex In 80/20 hydrogel in the dry state (**2a**–**c**).

Figure 6 displays SEM images comparing a neat dextran/inulin (Dex/In 80/20) hydrogel, synthesized in water, with its drug-loaded counterpart at magnifications of 150× and 200×. The microstructure of the neat xerogels exhibits a continuous, moderately rough surface. The absence of sharp interfaces or segregated domains indicates that modified dextran and inulin form a miscible matrix at the microscale, supporting the notion of their uniform physicochemical compatibility during hydrogel formation. Notably, the in situ incorporation of drug does not generate distinct morphological features that would allow direct visual identification in the SEM micrographs. While no discrete crystalline or particulate domains attributable to the drug are observed at these magnifications—indicating that the active compound is molecularly dispersed within the polysaccharide matrix—its incorporation still exerts a subtle but discernible influence on the drying-induced morphology. Compared with the neat Dex/In sample, the drug-loaded xerogel displays more fragmented and angular fracture surfaces, with sharper edges and plate-like domains that are absent in the unloaded network. These morphological features suggest that the dissolved drug alters the crosslinking and solidification behavior of the hydrogel during drying, increasing local brittleness and promoting the formation of more faceted fracture planes. Thus, although the drug cannot be visually identified as a separate phase, its incorporation clearly affects the structural organization of the dried matrix, leading to a more compact and fractured microstructure [24].

### 2.4. Results of Swelling Analysis

**Figure 7:**
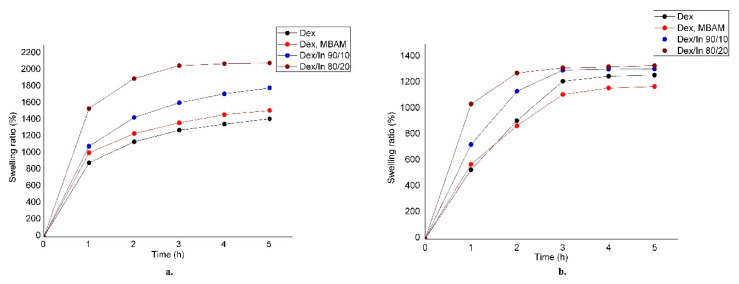
Swelling curves for hydrogels at: (**a**) pH 3 and (**b**) pH 6.

Figure 7 displays swelling curves at pH 3 and pH 6, revealing insights into network structure and a similar swelling pattern. A hydrogel prepared from neat dextran (Dex) and by applying a crosslinking agent, MBAM (Dex, MBAM), possesses similar swelling behavior. It is probably due to the short chain of the crosslinking agent, which does not significantly influence the architecture of the network. However, the inclusion of inulin in the hydrogel composition results in higher swelling ratios. This increased swelling is attributed to a lower crosslinking density within the hydrogel, consistent with the reduced ability of modified inulin, compared to modified dextran, to form a tightly crosslinked hydrogel network. It is consistent with the results of the methacrylation degree determination that dextran exhibits a higher degree of methacrylation than inulin. Consequently, hydrogels based on neat dextran contain a higher concentration of incorporated double bonds, which is directly proportional to the resulting crosslinking density. Dex hydrogel exhibits a very similar swelling behavior at both pH values, suggesting that pH variations have less influence on its intrinsic swelling properties. However, the addition of (MBAM) as a crosslinking agent introduces distinct differences in swelling, likely due to the pH-sensitive nature of its amide groups. Amides typically protonate under the highly acidic conditions, introducing a positive charge within the network, leading to electrostatic repulsion between biopolymer chains and an increase in swelling. Conversely, at higher pH values (like pH 6), these groups would be less protonated, reducing this repulsion and thus resulting in a lower swelling ability compared to their highly protonated state. This differential protonation of the crosslinker, even if subtle, can alter the effective crosslinking density or introduce localized charges that affect the overall swelling behavior. Even if it is slight, this differential protonation of the crosslinker can alter the effective crosslinking density or modify the overall swelling behavior by introducing localized charges. Statistical analysis of swelling analysis results is given in the Supplementary Materials (Table S1a,b).

### 2.5. Results of Mechanical Analysis

**Figure 8:**
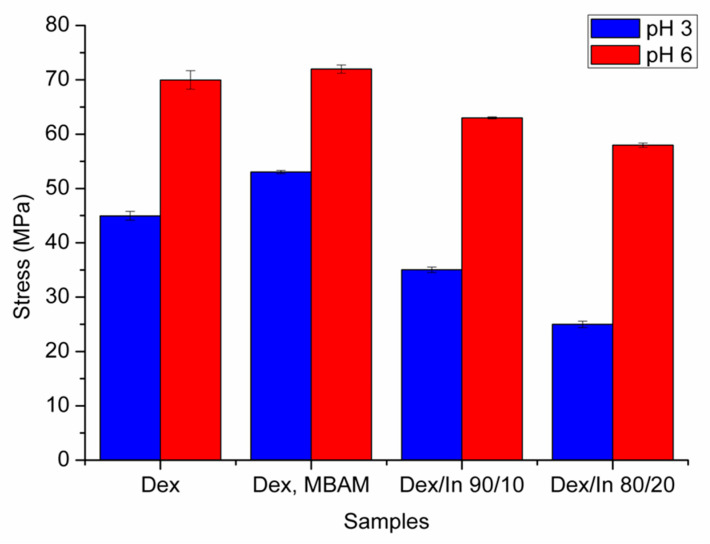
Stress (elastic moduli) values of granulated hydrogels in the equilibrium swelling state at pH 3 and pH 6.

Figure 8 represents the stress values, providing a direct measure of the granulated hydrogels’ resistance to shear forces. As the equilibrium swelling ratio increases, the hydrogel’s resistance to the shear force decreases. Higher elastic modulus values correspond to lower equilibrium swelling ratios, due to a higher crosslinking density and a greater ability to resist shear forces. Statistical analysis of mechanical analysis results is given in the Supplementary Materials (Table S2a,b). In comparison with the Dex–MA hydrogels prepared under UV irradiation as reported by Paoletti et al. [25], the maximum stress sustained before breakage is lower for the hydrogels tested in their granulated form. This outcome is expected, as the Dex–MA gels in the referenced study were obtained as compact, disc-shaped bulk structures with continuous network integrity, whereas the hydrogels in the present work were evaluated as granules. The lack of a unified monolithic form inherently reduces mechanical strength, leading to lower stress values at break.

### 2.6. Results of In Vitro Gastrointestinal Digestion (GID)

The results of digestion, as indicated by the enzymatic activity of enzymes from various parts of the digestive tract, are presented in Table 1. The primary results show a significantly higher percentage of active substance release in the intestinal region compared to the acidic environment of the gastric phase (stomach), which confirms the potential of hydrogels for targeted drug delivery in the lower gastrointestinal tract. Increasing the proportion of inulin in the hydrogel composition reduces the percentage of drug released in both the intestinal region and the stomach. This finding is attributed to the relatively poorer biodegradability of inulin compared to dextran under given in vitro conditions, thus emphasizing that the choice of carbohydrate biopolymer directly affects the kinetics of network degradation and, consequently, the efficacy and drug release profile. At the end of the procedure, the presence of residual gel was detected exclusively in samples containing inulin, amounting to 7.53 (Dex/In 90/10) and 16.24% (Dex/In 80/20), respectively, which can be attributed to the absence of inulinase activity during the intestinal phase. This behavior aligns with the chemical susceptibility of the respective polysaccharides: dextran contains glycosidic bonds that are more prone to acid-catalyzed hydrolysis in the gastric phase, whereas inulin remains largely resistant. Consequently, the inulin-rich domains within the network act as protective barriers, limiting the penetration and degradation of the dextran hydrogel matrix and thereby modulating the overall release kinetics. This highlights the critical influence of carbohydrate selection on the performance of hydrogels in drug delivery systems [20,21,22]. These findings can be reasonably extrapolated to the same carriers loaded with 5-fluorouracil (5-FU), given the close structural analogy between 5-FU and uracil, their comparable molecular dimensions, and their similar aqueous solubility profiles. Such physicochemical similarity suggests that 5-FU can be analogously encapsulated within these carriers, with the added advantage that 5-FU provides potent antitumor activity.

For the hydrogels that released uracil in the gastric phase (Dex and Dex, MBAM), the release behavior was successfully described using the Korsmeyer–Peppas model, with the kinetic parameters *k* and *n* listed in Table 2. The relatively low *k* value indicates that the intrinsic release rate is moderate and primarily governed by the structural characteristics of the Dex-based network, such as its degree of crosslinking, hydrophilicity, and internal porosity. In contrast, the notably high *n* value—greater than 1—reveals a super case-II transport mechanism, signifying that polymer relaxation, network rearrangement, and erosion predominantly dictate the release process [26]. In these systems, the release rate increases over time as the hydrogel matrix progressively undergoes fragmentation, rather than following simple Fickian diffusion. These results align with SEM findings, where uracil-loaded xerogels exhibit more angular and fragmented fracture features, indicative of increased brittleness and modified drying behavior. The obtained R^2^ values of 0.91 and 0.92 (Table 2) indicate a good level of agreement between the experimental release data and the model predictions. These coefficients of determination show that approximately 91 and 92% of the variability in drug release can be explained by the Korsmeyer–Peppas equation, confirming that this model provides a reliable description of the release kinetics.

### 2.7. Results of Encapsulation Efficiency (EE) Determination

Hydrogel samples exhibited similar encapsulation efficiency (EE) values (Table 3), ranging from 88.89% to 91.63%. The highest EE was obtained for the neat Dex, MBAM hydrogel, which is consistent with its enhanced network density and increased availability of interaction sites introduced by MBAM.

## 3. Conclusions

Based on the presented results, it can be concluded that a new, energy-efficient, and environmentally friendly process for synthesizing dextran and dextran/inulin hydrogels has been developed. Dextran and inulin, detected as biopolymers degradable only under the enzymes of the lower parts of the digestive tract, have been modified by glycidyl methacrylate under mild conditions (room temperature, DMSO solution). In the second step, hydrogels based on the modified biopolymers have been synthesized in a water solution in a few minutes, at room temperature, using MBAM as a crosslinking agent. Optimization of the composition by varying the inulin content (0–20 wt%) enabled precise tuning of the physicochemical properties. Increasing the inulin content led to the highest swelling capacity at both tested pH values (pH 3 and pH 6), which is directly related to the resulting lowest crosslink density. The inverse relationship between crosslink density and shear stress resistance was confirmed by the analysis of mechanical properties. Finally, in vitro digestion analysis of hydrogels with incorporated uracil proved the concept of dextran-based targeted drug delivery, while the increased proportion of inulin in the hydrogel composition resulted in reduced biodegradation throughout the digestive tract, highlighting the possibility of fine-tuning the degradation and release profile of the active compound by optimizing the hydrogel composition with a greater amount of dextran.

## 4. Materials and Methods

### 4.1. Materials

Dextran from *Leuconostoc* (Mr~40,000 g/mol, CAS: 9004-54-0), inulin from chicory (M_w_~500–3600 g/mol; CAS: 9005-80-5, free Glucose < 0.05%, by enzymatic assay; free fructose < 0.05% by enzymatic essay; ratio fructose:glucose ≥ 20:1), glycidyl methacrylate, and N,N′-Methylenebis(acrylamide) (MBAM), N,N,N’,N’-Tetramethylethylenediamine (TEMED), NaCl, NaHCO_3_, KCl, KH_2_PO_4_, MgCl_2_·6H_2_O, (NH_4_)_2_CO_3_, CaCl_2_·2H_2_O, Pefabloc^®^, dextranase, pepsin, bile salts, pancreatin, uracil (analytical grade) were provided by Sigma Aldrich (St. Louis, MO, USA). Potassium persulfate (PPS), NaOH, HCl, and 96% ethanol were supplied by CENTROHEM (Stara Pazova, Serbia). Dimethyl sulfoxide (DMSO) was procured from Merck KGaA (Darmstadt, Germany). Acetone was purchased from T.T.T. d.o.o. Sveta Nedelja (Sveta Nedelja, Croatia), while 4-dimethylaminopiridine was obtained from Thermo Fisher Scientific Inc. (Waltham, MA, USA). Buffer solutions with pH values of 3 and 6 were supplied from Reagecon (Shannon, Ireland).

### 4.2. Preparation of Hydrogels

**Figure 9:**
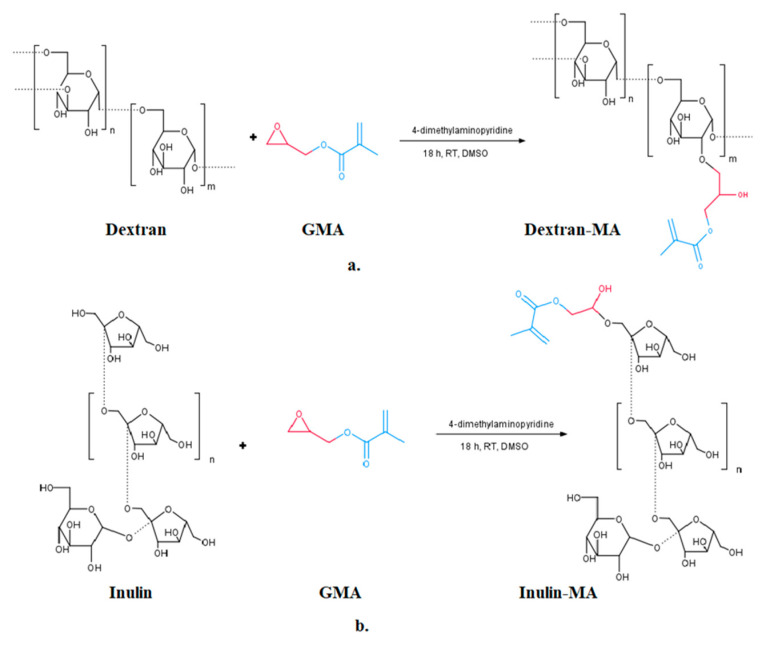
(**a**) the reaction modification of dextran, and (**b**) inulin.

Hydrogels were prepared using a two-step procedure. The first step implied modification of dextran and inulin using glycidyl methacrylate to incorporate double bonds into their structure (according to the modified procedure reported by De Smedt et al. (1995) [27]. The modification was performed by dissolving dextran and inulin (individually) in DMSO, at a concentration of 10 wt%, at 50 °C, with constant stirring. After complete dissolution of the biopolymers, 4-dimethylaminopiridine (7 wt% per biopolymer weight) was added as a catalyst. Stirring was continued until the catalyst had completely dissolved. Subsequently, the temperature was decreased to room temperature, and glycidyl methacrylate was added to the solutions in a 3:2 weight ratio with the biopolymer. The reaction was performed at room temperature for 18 h and stopped by the addition of acetone. The modified dextran and inulin were precipitated with acetone, purified with ethanol (1:3 weight ratio), and dried at 45 °C for 3 h. The scheme of the reaction modification of dextran and inulin is presented in Figure 9. For the preparation of hydrogels, modified dextran and inulin were dissolved in water (20 wt%) on a magnetic stirrer. After complete dissolution, 1 mL of MBAM water solution (0.04 g/mL), 1 mL of TEMED solution (0.027 g/mL) were added at continuous stirring in the solution of neat modified dextran and the solution of dextran and inulin in a ratio of 90/10 and 80/20. One sample based on neat dextran was prepared without a crosslinking agent, while the following steps were carried out in the same way as for the preparation of the rest three samples. After complete homogenization, as a final step, 1 mL of PPS solution (0.04 g/mL) was added. A few minutes after the addition of initiator PPS, hydrogels were formed. Table 4 gives an insight into neat hydrogel preparation and composition. Drug-loaded hydrogels were prepared by in situ incorporation of uracil by the addition of 0.02 g of uracil to the modified biopolymers’ water solution after the addition of 1 mL MBAM solution and 1 mL of TEMED solution. Complete homogenization at room temperature has provided conditions for the addition of 1 mL of PPS solution to initiate gel formation in a few minutes. Uracil was selected as a model drug due to its close structural similarity to the anticancer agent 5-fluorouracil and its well-established safety profile. This enables reliable evaluation of drug delivery systems under conditions that closely mimic the behavior of 5-fluorouracil, while avoiding the toxicity and strict handling requirements associated with chemotherapeutics. Uracil and 5-fluorouracil differ only by the substitution of a hydrogen atom with a fluorine atom at the C-5 position of the pyrimidine ring, resulting in comparable molecular size, polarity, and diffusion behavior in hydrophilic polymer networks such as dextran-based hydrogels. Accordingly, uracil serves as a non-toxic, safe, and cost-effective surrogate for preliminary investigations of encapsulation efficiency, swelling-controlled uptake, and degradation-triggered release without compromising the relevance of the obtained release trends. Chemical structures of both compounds are given in Figure S1a,b in the Supplementary Materials.

The degree of methacrylation was calculated as the difference between the saponification value (SV) and the aid value (AV). The acid value (AV) was determined according to the standard method ASTM D3644 [28]. Potentiometric titration of dextran methacrylate and inulin methacrylate, previously dissolved in distilled water, was performed using 0.1 N KOH. The AV was calculated according to the following Equation (1):

(1) $$AV=\frac{56.1\times c_{\mathrm{KOH}}\times V_{\mathrm{KOH}}}{m_{mb}}$$

The obtained acid value was 18 mg KOH g^−1^.

The saponification value (SV) was determined according to ISO 3657:2020 [29]. The method is based on the alkaline hydrolysis of ester groups and subsequent neutralization of excess KOH using a standard 0.1 mol dm^−3^ HCl solution. The SV was calculated using the following Equation (2):

(2) $$SV=\frac{56.1\times c_{\mathrm{HCl}}\times \left(V_{HCl0}-V_{\mathrm{HCl}}\right)}{m_{\mathrm{mb}}}$$

where V_HCl0_ is a volume of HCl used for neutralization of KOH solution (blank titration), and V_HCl_ is a volume of HCl used for neutralization of modified biopolymers (*m_mb_*) solution after treatment with KOH.

The obtained SV value was 396 mg KOH/g for Dextran-MA, and 264 mg KOH/g for In-MA.

Methacrylation degree, determined as the difference between SV and AV for Dex-MA, was found to be 378 mg KOH/g, and it relates to 1.2 hydroxy groups methacrylated with GMA. For the In-MA, the methacrylation degree was found to be 264 mg KOH/g, relating to the 0.81 hydroxy groups methacrylated with GMA.

### 4.3. Infrared Spectroscopy with Fourier Transformation (FTIR) Analysis

Shimadzu IRaffinity-1s Fourier Transform Infrared Spectrometer (Kyoto, Japan) operating in Attenuated Total Reflectance (ATR) mode (MIRacle 10 ATR-FTIR; Dia/ZnSe) was used to investigate the structure of modified biopolymers and hydrogels. 40 scans were averaged at a spectral resolution of 4 cm^−1^.

### 4.4. Differential Scanning Calorimetry (DSC) Analysis

Phase transitions and the successful modification of dextran and inulin were evaluated through Differential Scanning Calorimetry (DSC). Measurements were performed on a TA Instruments Q20 system (New Castle, DE, USA) under a nitrogen flow of 50 mL/min. Before analysis, the instrument underwent calibration for temperature and cell constant using indium reference samples, and for heat capacity (*C_p_*) using a sapphire crystal (both supplied by TA Instruments). Samples were subjected to a heating ramp from 25 to 220 °C at a rate of 10 °C/min. The melting temperature (*T_m_*), serving as an indicator of modification, was identified as the peak temperature. Glass transition temperatures (*T_g_*) were determined from the midpoint of the heat capacity change (*ΔC_p_*). All reported temperatures have a standard uncertainty of u(T) = 0.5 °C. Data interpretation and visualization were facilitated by the TA Analyzer software package (version 11.0).

### 4.5. Scanning Electron Microscopy (SEM) Analysis

The morphology of hydrogels has been investigated by a JEOL JSM-6390 scanning electron microscope (SEM), JEOL Ltd., Tokyo, Japan. Before imaging, samples were prepared with a conductive coating applied by a BALTEC SCD 005 device (BAL-TEC GmbH, Lübeck, Germany). Images were recorded at magnifications of 150× and 200×.

### 4.6. Analysis of Swelling Properties

The swelling ability of the hydrogels was investigated at a physiological temperature of 37 °C in buffer solutions of pH 3 and pH 6 for 5 h. This approach aimed to simulate the swelling conditions encountered throughout the digestive tract. The equilibrium swelling degree (ESD) was determined using Equation (3):

(3) $$ESD(\%)=\frac{m_{t}-m_{0}}{m_{0}}\cdot 100\%$$

where m_t_ represents the weight of the swollen gel at a specific time interval (1, 2, 3, 4, or 5 h), and m_0_ is the initial weight of the xerogel. Measurements were carried out in triplicate, and statistical analysis was added in the Supplementary Materials (Tables S1a and S2b).

### 4.7. Analysis of Mechanical Properties

**Figure 10:**
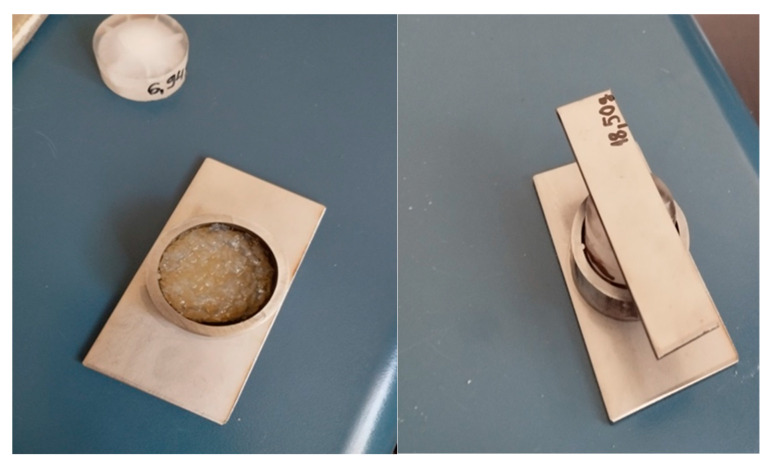
Experimental set-up for the mechanical testing.

Granulated xerogels, with a diameter between 1–2 mm, were prepared and weighed (0.3 g). This size fraction was determined by sequentially passing the material through a U.S. Standard Sieve No. 10 (2.00 mm nominal opening) and retaining it on a U.S. Standard Sieve No. 18 (1.00 mm nominal opening) [30]. The sized granulated xerogels were then immersed in buffer solutions maintained at two distinct pH values: pH 3.0 and pH 6.0. The immersion was continued for a period of 5 h at 37 °C, which was experimentally determined as the time required to achieve the maximum swelling ratio for the hydrogels under these specific conditions. Upon reaching their maximum swelling capacity, the fully swollen hydrogel granules were carefully removed from the buffer solutions (by colander, to remove the excess water) and immediately subjected to mechanical characterization using the experimental setup presented in Figure 10. This involved applying orthogonal shear stress using a specialized analyzer with a cylindrical probe (made from stainless steel, with defined roughness parameters); bottom plate with a diameter of 25 mm, Ra = 0.371; Rq = 0.485; Rz = 2.787; Rp = 0.645 µm; supporting ring Ra = 0.075; Rq = 0.105; Rz = 0.609; Rp = 0.381, Ra—arithmetic average roughness, Rq—root mean square roughness, Rz—average maximum height of the profile, Rp—maximum profile peak height. Roughness parameters were measured by using a Mitutoyo SJ-220 (Mitutoyo Corporation, Kawasaki, Japan) portable surface tester. The shear stress was incrementally applied until the point of hydrogel disintegration, marked by the rapid release of entrapped water. This method allowed for the determination of the hydrogel’s mechanical integrity and resistance to shear forces in the equilibrium swollen state. Measurements were conducted in triplicate, and average values and standard errors were presented in Figure 8. Statistical analysis was added in the Supplementary Materials (Table S2a,b). The granulated form of the hydrogels was selected for mechanical testing because it reflects their intended mode of oral administration, where the material would be encapsulated in hard gelatin capsules as free-flowing granules rather than as bulk gels.

### 4.8. Simulated In Vitro Gastrointestinal Digestion (GID)

Standardized static in vitro digestion was conducted according to the protocol previously described by Kostić et al. (2021) [31,32], with slight modifications in the simulated intestinal phase of digestion. Also, since the hydrogels are predicted to be digested as capsules, the analysis of the oral phase was omitted because the formulation is intended for the application as a hard-gelatin capsule filled with xerogel granules. In this dosage form, the material is swallowed immediately and does not remain in the oral cavity; therefore, it does not undergo oral residence, disintegration, or interaction with saliva before being released in the stomach. Consequently, inclusion of the oral digestion phase is not relevant for this pharmaceutical form. Three experimental groups in triplicate were created. The digestion started (gastric phase of digestion) with mixing 0.25 g of xerogel (equivalent to 2.5 g of rehydrated sample) incorporated in gelatine capsules (26.1 mm) with 7.5 mL of simulated gastric fluid (SGF), 1.6 mL of pepsin solution (25,000 U/mL), and 5 μL of 0.3 M CaCl_2_. The pH value was adjusted to 2.0 using 1 M HCl, and distilled H_2_O was used to adjust the volume of the mixture to 20 mL. Incubation of samples during the gastric phase was conducted at 37 °C for 2 h with continuous shaking (250 rpm). In the next intestinal phase of digestion gastric chyme was mixed with 11 mL of simulated intestinal fluid (SIF), 5 mL of pancreatin solution (800 U/mL trypsin activity), 0.2 mL of dextranase (100 U/mL) 2.5 mL of 160 mM bile salts, 40 μL of 0.3 M CaCl_2_, pH was adjusted to 7.0 and supplemented with distilled H_2_O to achieve volume of 40 mL. For the intestinal phase, incubation lasted for 2 h at 37 °C with continuous shaking (250 rpm). The composition of SGF and SIF is prepared according to the protocol INFOGEST [30] presented in Table 5. Afterwards, the enzyme reactions were stopped using Pefabloc^®^. The sample group counting only empty hydrogels was prepared as a control for digested uracil-containing hydrogels, as previously described was immediately mixed with all enzymes and solutions required for in vitro digestion, supplemented with Pefabloc^®^ to inhibit enzyme reactions instantaneously. Also, the third group contained a digestive cocktail control—2.5 g of distilled water (instead of the sample) was immediately mixed with all digestive enzymes and solutions, as well as with Pefabloc^®^ to stop the enzyme reaction. Upon completion of treatments, samples were centrifuged at 10,000 rpm for 20 min at 4 °C, supernatants were kept, and their volume was accurately measured and stored at −20 °C for further analysis. The procedures were carried out in triplicate, and the statistical analysis was added to the Supplementary Materials (Table S3a,b).

Release profile of uracil has been determined for the Dex and Dex, MBAM hydrogels, whose releasing begin in the stomach. For the determination of kinetics, the fraction of uracil released from the Dex, MBAM formulation as a function of time was analyzed using the empirical Korsmeyer–Peppas model (Equation (4)) [26]:

(4) $$\frac{M_{t}}{M_{\infty }}=kt^{n}$$

where M_t_ is the percentage of drug released at time t and $M_{\infty }$ was taken as 100% release. *k* is kinetic constant that reflects the structural and geometric properties of the drug delivery system; *n* is release exponent that characterizes the mechanism by which the drug is released. The data were linearized according to Equation (5):

(5) $$log\left(\frac{M_{t}}{M_{\infty }}\right)=logk+n\;log\;t$$

And fitted by linear regression using the time points from 1 to 4 h.

### 4.9. Determination of Uracil Concentration in Digestate by UV/VIS Spectrophotometry

The UV/VIS spectrophotometry method for uracil determination was modified from Khajehsharifi and Soleimanzadegan (2013) [32,33]. An Agilent BioTek EPOCH 2 UV-Vis spectrophotometer (Agilent Technologies, Santa Clara, CA, USA) photodiode array spectrophotometer with GEN5 Software version 3.11 (Agilent BioTek, Santa Clara, CA, USA) and computer, equipped with a 1 cm path-length quartz cell, was used for UV-VIS spectra determination. Approximately 25 mg of 25 mL of distilled water by sonication for 5 min. The final volume was adjusted to 100 μg/mL. A series of solutions was prepared for the determination of the standard calibration curve. The standard solutions had the following concentrations: 100, 50, 25, 12.5, 6.25, and 3.125 μg/mL. The linear dynamic range for each component was determined by regressing the absorbance at the corresponding λ_max_ versus the analyte concentration. Using a standard stock solution (100 μg/mL), the wavelength for analysis of uracil was selected from the UV-spectrum. To find out the λ_max_, the standard stock solution was scanned in the UV range between 200–400 nm. The absorbance maxima (λ_max_) were found at 230 nm against distilled water. For the determination of uracil in digestate, the absorbance was read against a blank digestate solution containing empty hydrogel for each specific hydrogel mixture.

### 4.10. Determination of Encapsulation Efficiency

For the determination of encapsulation efficiency, the modified method described by Calina et al. [33,34,35] was applied. Encapsulation efficiency (EE) as a measure of the ability of the hydrogel to incorporate active compounds was determined according to Equation (4):

$$EE\;\left(\%\right)=\frac{m_{t}}{m_{i}}\cdot 100\%$$

where $m_{t}$ represents the total amount of uracil incorporated into the xerogel, and $m_{i}$ denotes the initial mass of uracil added during hydrogel preparation (0.2 g per g of dry xerogel). The total incorporated uracil was determined using a two-step extraction–quantification protocol. In the first step, xerogel samples were immersed in DMSO for 12 h to allow complete release of uracil, followed by centrifugation and filtration to remove insoluble residues. In the second step, the concentration of uracil in the obtained supernatants was quantified using UV–Vis spectrophotometry (Agilent BioTek EPOCH 2). A calibration curve was constructed using standard uracil solutions prepared in DMSO to ensure accurate quantification. All measurements were performed in triplicate, and the results are summarized in Table 2.

### 4.11. Statistical Analysis

The experiments were done in triplicates. All values are expressed as means ± standard deviation. Mean values of the amounts of released uracil from prepared hydrogels (digestion) and Encapsulation efficiency (EE) were compared by the analysis of variance (one-way ANOVA) followed by Tukey’s HSD post hoc test for mean differences testing (SPSS Statistica 20, IBM Corporation, Armonk, NY, USA). Differences were considered significant at *p* < 0.05.

## Figures and Tables

**Table 1:** The amounts of released uracil from prepared hydrogels due to the biodegradation under the enzymes presented in the gastric and intestinal phases.

Uracil-Loaded Hydrogels	Gastric Phase Release, µg/mL	Intestinal Phase Release, µg/mL	Gastric Phase Release, %	Intestinal Phase Release, %	Total Released Amount of Drug, %
Dex	19.46 ±0.57 ^c^	80.21 ± 0.72 ^c^	16.57 ± 0.46 ^c^	64.17 ± 0.57 ^c^	80.74 ± 0.73 ^c^
Dex, MBAM	16.81 ± 0.28 ^b^	87.29 ± 0.51 ^d^	13.45 ± 0.23 ^b^	69.84 ± 2.07 ^d^	83.29 ± 2.08 ^c^
Dex/In 90/10	0 ± 0.00 ^a^	75.47 ± 1.85 ^b^	0 ± 0.00 ^a^	60.37 ± 2.08 ^b^	60.37 ± 2.08 ^b^
Dex/In 80/20	0 ± 0.00 ^a^	59.71 ± 1.85 ^a^	0 ± 0.00 ^a^	47.76 ± 2.49 ^a^	47.76 ± 2.49 ^a^

The maximum amount that can be released is 125 μg/mL, calculated on a content of 20 mg per g of dry carrier, and 0.25 g of dry material. ^a,b,c,d^—groups sharing a same letter are not significantly different from each other, while the groups with different letters are significantly different.

**Table 2:** Korsmeyer–Peppas parameters for neat dextran hydrogels.

Uracil-Loaded Hydrogels	*n*	*k*	R^2^
Dex	2.66	0.03	0.92
Dex, MBAM	1.59	0.09	0.91

**Table 3:** Encapsulation efficiency (EE) of hydrogel samples.

Uracil-Loaded Hydrogels	Encapsulation Efficiency (%)
Dex	90.10 ± 1.45 ^a^
Dex, MBAM	91.63 ± 1.16 ^a^
Dex/In 90/10	89.98 ± 0.45 ^a^
Dex/In 80/20	88.89 ± 1.59 ^a^

Superscript ^a^—groups are not significantly different from each other.

**Table 4:** Initial amounts of compounds for the preparation of the hydrogel.

Samples	Dextran–MA ^1^, g	Inulin–MA ^2^, g
Dex	1	0
Dex, MBAM	1	0
Dex/In 90/10	0.9	0.1
Dex/In 80/20	0.8	0.2

^1^ Dextran–MA = methacrylated dextran, ^2^ Inulin–MA = methacrylated inulin.

**Table 5:** Composition of stock solutions and simulated digestion fluids.

			SGF	SIF
			pH 3.0	pH 7.0
Compound	Stock Conc.		Conc. in SGF	Conc. in SIF
	g/L	mol/L	mmol/L	mmol/L
KCl	37.3	0.5	6.9	6.8
KH_2_PO_4_	68	0.5	0.9	0.8
NaHCO_3_	84	1	25	85
NaCl	117	2	47.2	38.4
MgCl_2_·6H_2_O	30.5	0.15	0.1	0.33
(NH_4_)_2_CO_3_	48	0.5	0.5	-
NaOH	-	1	-	8.4
HCl	-	6	15.6	-
CaCl_2_ 2H_2_O	44.1	0.3	0.075	0.3

SGF-simulated gastric fluid, SIF-simulated intestinal fluid. All simulated fluids were prepared as 1.25× concentrates since the subsequent addition of enzymes, bile salts, Ca^2+^ solution, and water will result in the correct concentration of each compound in the final digestion mixture. Concentrations refer to the final digestion mixture; CaCl_2_·2H_2_O was added separately.

## Data Availability

The original contributions presented in this study are included in the article/Supplementary Materials. Further inquiries can be directed to the corresponding author.

## Supplementary Materials

The following supporting information can be downloaded at: https://www.mdpi.com/article/10.3390/gels11121011/s1, Figure S1: Structure of: a. uracil, b. 5-fluorouracil; Table S1a: Statistical analysis of the ESR values at pH 3; Table S1b: Statistical analysis of the ESR values at pH 6; Table S2a. Statistical analysis of the mechanical strength values at pH 3; Table S2b. Statistical analysis of the mechanical strength values at pH 6; Table S3a. Statistical analysis of the digestion of capsulated xerogels in the gastric phase expressed in concentration and in percent; Table S3b. Statistical analysis of the digestion of capsulated xerogels in the intestinal phase expressed in concentration and in percent; Table S4. Statistical analysis of the digestion of capsulated xerogels in the intestinal phase expressed in concentration and in percent.

## Abbreviations

The following abbreviations are used in this manuscript:

DMF	Dimethylformamide
MA	Methacrylic acid
IN-MA	Inulin metahcrylate
BMAAB	bis(methacryloylamino)azobenzene
HEMA	2-hydroxyethyl methacrylate
Dex–MA	Dextran-methacrylate
EPR	Enhanced permeability and retention
FTIR	Infrared spectroscopy with Fourier transformation
DSC	Differential scanning calorimetry
DMSO	Dimethyl sulfoxide
MBAM	*N, N*′-Methylenebis (acrylamide)
TEMED	*N,N,N’,N’*-Tetramethylethylenediamine
PPS	Potassium persulfate
SIF	Simulated intestinal fluid
SGF	Simulated gastric fluid
EE	Encapsulation efficiency
