# Supplementary material for: The Influence of Synthesis Parameters on the Properties of Dextran-Based Hydrogels for Colon-Targeted Antitumor Drug Delivery Part I: Room Temperature Synthesis of Dextran/Inulin Hydrogels for Colon-Targeted Antitumor Drug Delivery
Source: Gels. 2025 Dec 16;11(12):1011. doi: 10.3390/gels11121011 (PMC12732961; doi:10.3390/gels11121011)
Supplement: Supplementary file 1 [file gels-11-01011-s001.zip › gels-4013374-supplementary.pdf]

The Influence of Synthesis Parameters on the Properties of Dextran-Based Hydrogels for Colon-Targeted Antitumor Drug Delivery Part I: Room Temperature Synthesis of Dextran/Inulin Hydrogels for Colon-Targeted Antitumor Drug Delivery

Tamara Erceg [1,*], Miloš Radosavljević [1], Milorad Miljić [2], Aleksandra Cvetanović Kljakić [1], Sebastian Baloš [3], Katarina Mišković Špoljarić [4], Ivan Ćorić [4], Ljubica Glavaš-Obrovac [4] and Aleksandra Torbica [2]

[1] Faculty of Technology Novi Sad, University of Novi Sad, Bulevar Cara Lazara 1, 21000 Novi Sad, Serbia; milos1506@gmail.com (M.R.); a.c.istrazivac@gmail.com (A.C.-K.)
[2] Institute of Food Technology in Novi Sad, University of Novi Sad, Bulevar Cara Lazara 1, 21000 Novi Sad, Serbia; milorad.miljic@fins.uns.ac.rs (M.M.); aleksandra.torbica@fins.uns.ac.rs (A.T.)
[3] Faculty of Technical Sciences, University of Novi Sad, Trg Dositeja Obradovića 6, 21000 Novi Sad, Serbia; sebab@uns.ac.rs
[4] Faculty of Medicine, Josip Juraj Strossmayer University of Osijek, Josipa Huttlera 4, 31000 Osijek, Croatia; kmiskovic@mefos.hr (K.M.Š.); icoric@mefos.hr (I.Ć.); lgobrovac@mefos.hr (L.G.-O.)
* Correspondence: tamara.erceg@uns.ac.rs

Supplementary Materials

Swelling properties

Table S1a. Statistical analysis of the ESR values at pH 3.

Samples	Mean	Median	Standard Error	Standard Deviation	Range	Confidence Level(95.0%)
**Dex**	1508.56	1510.88	3.37	5.84	10.97	14.51
**Dex, MBAM**	1456.47	1455.54	3.17	5.51	10.9	13.68
**Dex/In 90/10**	1742.15	1739.16	4.03	6.99	12.98	17.36
**Dex/In 80/20**	2091.38	2088.1	4.91	8.49	16.02	21.11

Table S1b. Statistical analysis of the ESR values at pH 6.

Samples	Mean	Median	Standard Error	Standard Deviation	Range	Confidence Level(95.0%)
**Dex**	1262.74	1260.37	3.13	5.42	10.04	14.48
**Dex, MBAM**	1175.25	1174.38	3.17	5.49	10.89	13.66
**Dex/In 90/10**	1310	1311.2	3.53	6.12	12.05	15.20
**Dex/In 80/20**	1335.82	1335.92	1.79	3.11	6.21	7.72

Mechanical properties

Table S2a. Statistical analysis of the mechanical strength values at pH 3.

Samples	Mean	Median	Standard Error	Standard Deviation	Range	Confidence Level(95.0%)
**Dex**	44.97	44.9	0.81	1.40	2.8	3.48
**Dex, MBAM**	53.03	53.1	0.29	0.50	1	1.25
**Dex/In 90/10**	35.03	35.2	0.49	0.86	1.7	2.14
**Dex/In 80/20**	25	25	0.58	1	2	2.48

Table S2b. Statistical analysis of the mechanical strength values at pH 6.

Samples	Mean	Median	Standard Error	Standard Deviation	Range	Confidence Level(95.0%)
**Dex**	69.97	69.2	1.69	2.93	5.7	7.27
**Dex, MBAM**	71.97	71.6	0.76	1.31	2.54	3.25
**Dex/In 90/10**	62.98	63.15	0.19	0.34	0.6	0.82
**Dex/In 80/20**	57.97	58.2	0.39	0.68	1.3	1.69

Results of in vitro gastrointestinal digestion (GID)

Table S3a. Statistical analysis of the digestion of capsulated xerogels in the gastric phase expressed in concentration and in percent.

Samples	Mean	Median	Standard Error	Standard Deviation	Range	Confidence Level(95.0%)
**Dex**	19.46; 16.57	19.23; 16.38	0.33; 0.27	0.57; 0.46	1.08; 0.86	1.47; 1.14
**Dex, MBAM**	16.81; 13.45	16.79; 13.41	0.11; 0.13	0.28; 0.23	0.56; 0.46	0.37; 0.56
**Dex/In 90/10**	0	0	0	0	0	0
**Dex/In 80/20**	0	0	0	0	0	0

Table S3b. Statistical analysis of the digestion of capsulated xerogels in the intestinal phase expressed in concentration and in percent.

Samples	Mean	Median	Standard Error	Standard Deviation	Range	Confidence Level(95.0%)
**Dex**	80.21; 64.17	80.11; 64.08	0.42; 0.33	0.72; 0.57	1.43; 1.43	1.79; 1.43
**Dex, MBAM**	87.29; 69.84	87.29; 70.45	0.29; 1.19	0.51; 2.07	1.02; 4.00	1.27; 5.14
**Dex/In 90/10**	75.47; 60.37	75.48; 59.87	1.07; 1.19	1.85; 2.07	3.71; 4.06	4.61; 5.15
**Dex/In 80/20**	59.71; 47.76	60.14; 46.84	1.06; 1.44	1.85; 2.49	3.62; 4.73	4.59; 6.20

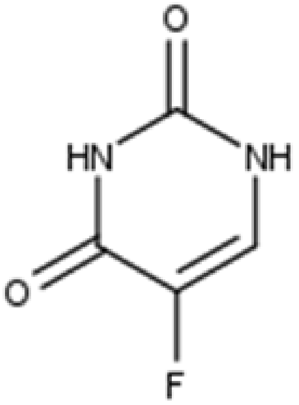

a.

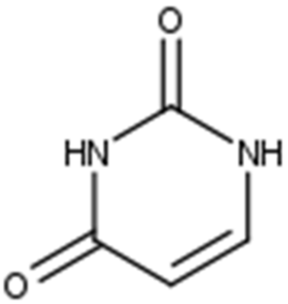

b.

Figure S1. Structure of: a. uracil, b. 5-fluorouracil.

Table S4. Statistical analysis of the digestion of capsulated xerogels in the intestinal phase expressed in concentration and in percent.

Samples	Mean	Median	Standard Error	Standard Deviation	Range	Confidence Level(95.0%)
**Dex**	90.10	90.30	0.84	1.45	2.89	3.61
**Dex, MBAM**	91.63	91.67	0.67	1.16	2.33	2.89
**Dex/In 90/10**	89.98	89.78	0.26	0.45	0.83	1.12
**Dex/In 80/20**	88.89	88.45	0.92	1.59	3.09	3.95
